# Failed attempts at experimental transplantation and transmission of nocturnally-periodic simian *Loa *from monkey to man

**DOI:** 10.1186/1475-2883-3-5

**Published:** 2004-07-29

**Authors:** BOL Duke

**Affiliations:** 1 River Blindness Foundation, 2 Hillside, Lancaster LA1 1YH, UK

## Abstract

This paper describes unsuccessful attempts to induce a nocturnally-periodic infection with simian *Loa *in a human volunteer (the author of this paper) by means of 1. Transplanting adult simian *Loa *worms from a wild drill (*Mandrillus leucophaeus*) to man; and 2. Infecting the same volunteer by sub-cutaneous inoculation with infective larvae of simian *Loa *from a laboratory-bred, experimentally infected *Chrysops silacea*.

## Findings

In the mid 1950s, one of the main lines of research followed by the Helminthiasis Research Unit (HRU), at Kumba in the then British Cameroons was to establish the relationship between human infections with *Loa loa *and the highly similar *Loa *parasites which were found in several species of local monkey, especially in the drill (*Mandrillus leucophaeus*) but also, less frequently, in several long-tailed monkey species, *Cercopithecus nictitans martini*, *C. mona mona *and *C. preussi*.

Observations on 109 young drills (found to be free from natural *Loa *infection after a quarantine period of 6–8 months in screened cages), which were experimentally infected by transplantation of live adult simian *Loa *worms extracted from wild drills shot in the local forests, revealed that the parasites exhibited a nocturnal microfilarial periodicity with microfilariae (mf) that were significantly longer than those of human *L. loa*.

By contrast, drills which had been infected by inoculation of infective larvae from the day-biting *Chrysops silacea *or *C. dimidiata*, that had been experimentally infected 10 days previously by feeding on the blood of *Loa*-infected human volunteers, demonstrated diurnally-periodic microfilarial infections, whose parasites (both adult worms and mf) were of shorter length than the normal wild monkey parasites [1].

Further studies on the biting habits of local *Chrysops *species revealed that in nature the diurnally periodic human *Loa loa *was transmitted among humans by the day-biting species *C. silacea *and *C. dimidiata*, whereas the nocturnally periodic simian parasite was being transmitted among monkeys by two forest-canopy-dwelling species, *C. langi *and *C. centurionis*, both of which bite in the forest canopy at night and are presumed to feed mainly on sleeping monkeys [2].

No naturally-acquired diurnally periodic microfilarial infection was seen in 21 wild drills, the periodicities of whose adult worms were examined by transplantation into uninfected animals; despite the fact that male and female worms of human and simian *Loa *were quite capable, when transplanted together under experimental conditions into uninfected drills, of inter-breeding and producing microfilariae of intermediate dimensions and periodicity [3]. (This is not to say that under natural conditions diurnally-periodic worms of human *Loa *are never transmitted to monkeys, but such events, if they do occur, appear to be rare).

Later, Belgian workers in the Mayumbe District in the south-western part of the Democratic Republic of the Congo (DRC), commented on the local human *Loa *infections in that area being particularly liable to give rise to cases of *Loa*-encephalopathy after treatment with diethylcarbamazine citrate (DEC). They also noted that out of 547 patients from this area, whose blood films were examined both by day and night, 197 showed *Loa *mf only by day, 322 showed them by day and by night; and 16 showed mf only at night. Although the latter cases, with a complete reversal of the normal periodicity, showed only light microfilarial loads (12 cases with 1–5 mf per examination, and 4 cases with 6–9 mf per examination), among those persons who showed *Loa *mf by day and by night, 62 showed microfilarial concentrations that were nearly as high by night as by day and 10 showed more mf by night than by day (two of them showing 500 – 1,000 mf by night as compared with 300 – 500 mf by day) [4-6]. As some of the cases of loiasis from Mayumbe were abnormal in displaying a primarily nocturnal periodicity of the microfilariae, it is possible that the local strain of *Loa *responsible for them may be closely related to the simian parasite.

Recently in the Republic of Cameroon, cases of *Loa*-encephalopathy have been reported following mass treatment with ivermectin by the African Programme for Onchocerciasis Control (APOC) in areas where loiasis is co-endemic with onchocerciasis [7-11]. A remarkable clustering of many of these cases was found in the Lékié Division, a forest and forest/savannah mosaic area some 80 km from the capital, Yaoundé. So far, the reason for this clustering has not become apparent but the occurrence of these cases of *Loa*-encephalopathy has had a deleterious effect on the popularity of the APOC campaign in that area [12]. Furthermore, at the end of 2003 in the Mayumbe area of the DRC, some 100,000 persons were treated with a standard single dose of ivermectin distributed as part of the activities of APOC, and 41 cases of serious adverse reactions (SAEs) were reported, of which 14 were fatal despite appropriate management of the patients. This is an incidence rate even higher than that reported in Lékié Division of Cameroon and has led to the establishment of a commission to examine the matter (Dr B. Thylefors, *personal communication*).

The patho-biological reasons for the occurrence of *Loa*-encephalopathy following treatment with DEC or with ivermectin, mainly seen in patients heavily infected with *Loa *microfilariae, are not well understood, and co-factors may exist that account for the fact that some patients do not develop SAEs despite having high *Loa *microfilaraemia. Experimental work by Dr Samuel Wanji using an animal model is currently trying to reproduce heavy microfilaraemic infections of human *L. loa *in experimentally infected monkeys (mainly *Mandrillus *spp) and to investigate the biochemical and pathological changes that accompany the development of any *Loa*-encephalopathy following ivermectin treatment.

In the original work at the HRU, Kumba, where it was relatively easy to infect young drills experimentally (either by inoculation of infective larvae or by transplantation of adult worms) with either the nocturnally-periodic simian *Loa *parasite or with the diurnally-periodic human parasite, it was obviously far more difficult to determine whether the nocturnally-periodic simian parasite could be transferred to man. Nevertheless, at that time, before the discovery of the potentially deadly viruses such as Ebola, Marburg and HIV that are believed to originate from monkeys, attempts to infect a human (the author) experimentally with a simian strain of the *Loa *parasite were undertaken. Today, such experiments would not only be viewed as unethical but also as potentially life-threatening.

In 1954 and 1955, the author (who at that time had no signs or symptoms of loiasis and who was not taking any medication, apart from 200 mg proguanil (Paludrine) daily as a prophylactic for malaria), took part in two such experiments, which have not been previously published but are relevant in the light of the localised occurrence of *Loa*-encephalopathy in some individuals following treatment with ivermectin. These attempts at experimental infection of a human being with simian *Loa *are as follows:

In July 1954, a large male drill, which had been shot in the forest near Kumba some 3–4 hours previously, was brought into the laboratory by the Unit's hunter. It was immediately dissected and a total of seven male and fifteen female mature simian *Loa *worms, all alive, undamaged and motile, were collected from the subcutaneous and intermuscular tissues. The worms were placed in sterile normal saline solution, along with a small quantity of merthiolate, in the same manner that had been used previously to transplant adult simian *Loa *worms successfully into other monkeys. Two and a half hours later, 12 of these live, motile, adult female worms and five males were inserted, under local anaesthesia, into the upper, anterior part of the right thigh of the author by the Medical Officer-in-Charge of the Kumba Medical Field Unit. After making a 3-inch, longitudinal incision through the skin and the superficial fascia, the simian *Loa *worms were inserted, some into the sub-cutaneous tissue and others under the deep fascia of the *rectus femoris *muscle. The fascial layers were then sewn up, the skin closed with nylon sutures, and intramuscular penicillin was given to counteract infection.

Over the ensuing week the transplant area became considerably swollen and painful over an area of approximately 6–8 × 5–6 inches and over the following month it itched frequently. Day and night blood films (50 cu. mm) were taken once a week over a period of six months, and thereafter fortnightly for the next six months, but none of them detected any microfilariae. No *Loa *worm(s) appeared under the skin or crossing the eye, nor did any *Loa *mf appear in the peripheral blood over the next 46 years. (It is possible that all the worms died fairly soon after being inserted, or it may be that they remained alive but failed to produce a detectable microfilarial infection).

Eighteen months after the transplant, the author also injected himself subcutaneously in the left thigh with 35 live, motile, infective larvae of simian *Loa*, which had been dissected out in normal saline from a laboratory-bred female *Chrysops silacea *that had taken a blood-meal 10 days previously from a captive drill infected with the nocturnally-periodic simian strain of *Loa*. Previous experimental work with monkeys had shown that this method of infection was more effective than trying to induce a *Chrysops *containing infective larvae of *Loa *to feed on, and thus transmit infective *Loa *larvae to, an uninfected person. There was a slight reddening and itching of the skin in the area around the injection site over the following week but otherwise no papular eruption or other reaction developed. No *Loa *microfilariae were detected in the peripheral blood by day or by night over the ensuing eight years; nor, over the same period was any skin reaction seen that could have been attributed to the death of L3 or subsequent stages of *L. loa *dying in or under the skin, as were reported subsequently when infective larvae of *L. loa *from the bite of an experimentally-infected *Chrysops silacea *were subsequently killed by dosage with diethylcarbamazine citrate (DEC) used as a chemoprophylactic [13].

Both these attempts failed to infect a healthy human volunteer with the nocturnally-periodic simian strain of *Loa*. Obviously the failure to infect a single individual must be interpreted with caution. Nevertheless these observations are worth recording especially now that research is in progress to try and ascertain:

1. The factors leading to the localised occurrence of *Loa*-encephalopathy in certain areas of central Africa, where ivermectin is now being used currently for the control of onchocerciasis, or where in the past it has occurred following treatment with diethylcarbamazine; and

2. The biochemical and histological changes that may be associated with a *Loa*-encephalopathy if it can be induced in drills heavily infected with *Loa *mf when treated with ivermectin.

## Competing Interests

None declared.

## Authors' contributions

Brian Duke was sole author
